# SHP2 is essential for the progesterone-promoted proliferation and migration in breast cancer cell lines

**DOI:** 10.3389/fendo.2025.1523589

**Published:** 2025-02-10

**Authors:** Hui-Chen Wang, Wen-Sen Lee

**Affiliations:** 1 Graduate Institute of Medical Sciences, College of Medicine, Taipei Medical University, Taipei, Taiwan; 2 College of Medicine, National Taiwan University, Taipei, Taiwan; 3 Cancer Research Center, Taipei Medical University Hospital, Taipei, Taiwan; 4 Cell Physiology and Molecular Image Research Center, Wan Fang Hospital, Taipei Medical University, Taipei, Taiwan; 5 Department of Physiology, School of Medicine, Tzu Chi University, Hualien, Taiwan

**Keywords:** breast cancer cell lines, Csk, cSrc, p-cSrcY416, p-cSrcY527, SHP2

## Abstract

**Introduction:**

We previously demonstrated that progesterone (P4) can promote breast cancer cell proliferation and migration through activating the P4 receptor (PR)/cSrc-mediated signaling pathway. It has been suggested that high level of Src homology region 2 domain-containing phosphatase-2 (SHP2) might be involved in breast oncogenesis. This study aimed to investigate whether SHP2 is involved in the P4-mediated cSrc activation in breast cancer cells.

**Methods:**

T47D, MCF-7 and BT-483 breast cancer cell lines were used in this study. Cell proliferation and migration were examined using MTT technique and wound healing assay, respectively. Immunoprecipitation assay and Western blot analysis were performed to evaluate protein-protein interaction and protein expression, respectively. Small interfering RNA (siRNA) technique was used to knock down protein expression.

**Results:**

Knockdown of SHP2 expression abolished the P4-promoted cell proliferation and migration in T47D, MCF and BT-483 cell lines, suggesting that presence of SHP2 is essential for the P4-increased proliferation and migration of breast cancer cell lines. P4 (50 nM) treatment increased the complex formations of PR-cSrc-SHP2-caveolin-1, SHP2-p140Cap, and SHP2-Csk, and the level of p-cSrcY416 (activated form of cSrc). However, knockdown of SHP2 expression increased the complex formations of PR-cSrc-caveolin-1-Csk-p140Cap and the levels of p-caveolin-1, p-Csk and p-cSrcY527 (inactivated form of cSrc).

**Discussion:**

Our data suggest that SHP2 can bind to cSrc-negative regulatory proteins (p140Cap and Csk), hence preventing the interaction between cSrc and cSrc-negative regulatory proteins, leading to decreased phosphorylation of cSrc Y527 and prolonged cSrc activation. These findings highlight the role of SHP2 in the P4-promoted breast cancer cell proliferation and migration.

## 1 Introduction

In the developed countries, breast cancer is the most common cancer diagnosed and the most invasive cancer in women. The survival rates of breast cancer have increased largely due to a better understanding of the disease, earlier detection and better treatment.

Female sex hormones have been indicated as the risk factors for breast cancer development (1). Exposure of P4 and progestin has been recognized as instrumental in the breast cancer risk associated with exposure to the female sex hormones (2). Traditionally, P4 has been thought to regulate biological effects through binding to its nuclear receptors, PR-A or PR-B, to alter gene transcription. Lately, a growing body of evidence suggests that P4 could bind to the PR located in the cytoplasm or membrane to initiate a quick acting non-genomic signaling cascade. We previously showed that P4 increased migration in arterial smooth muscle cells through a cSrc-mediated non-genomic pathway (3). The rapid action of P4-induced cSrc activation has been implicated in the regulation of cell cycle and migration (3, 4). Moreover, it has been indicated that physiological levels of P4 might increase migration (5) and proliferation (6–8) of breast cancer cells via an extra-nuclear signaling pathway. We previously also demonstrated that P4 promoted breast cancer cell proliferation (9) and migration (10) through activating the P4 receptor (PR)/cSrc-mediated signaling pathways. However, the molecular mechanism underlying P4-induced cSrc activation is still unclear.

SHP2, encoded by the *PTPN11* gene, is a pro-migratory signal. SHP2 activates Src family kinases and downstream targets, hence enhancing cell invasion and migration (11). Experimental and clinical data have indicated that high levels of SHP2 mRNA correlate with poor progression-free survival and overall survival in lung adenocarcinomas (12) and SHP2 promotes tumor progression in many types of cancer including breast cancer (13). It has been reported that SHP2 can function as a positive regulator in cSrc signaling by interfering with the Csk-caveolin-1 complex formation in the H_2_O_2_-treated astrocytes (14). Knockdown of SHP2 in established breast cancers reduces their growth and metastasis (15). Moreover, reducing SHP2 by small interference RNA and adenovirus-mediated expression of a catalytically inactive mutant of SHP2 decreased cSrc activation (16). It has been demonstrated that transcription factors PR and CREB1 bind to the PTPN11 promoter to regulate the expression of SHP2 in response to decidual signals (17). However, whether SHP2 contributes to the P4-promoted breast cancer cell proliferation and migration is still unknown.

In the present study, we investigated the possible role of SHP2 in the P4-promoted breast cancer cell proliferation and migration. The experimental findings reported below highlight certain molecular mechanisms underlying P4-promoted breast cancer cell proliferation and migration.

## 2 Materials and methods

### 2.1 Cell culture

Three breast cancer cell lines (BT-483, MCF-7 and T47D) used in this study were purchased from the American Type Culture Collection/Bioresource Collection and Research Center (BCRC) (Taiwan), where the STR-PCR profiles were performed. The breast cancer cell lines were cultured as previously described (18). Briefly, breast cancer cell lines were grown in the medium containing Dulbecco’s Modified Eagle Medium (DMEM), 10% fetal bovine serum (FBS), penicillin (100 U/mL) and streptomycin (100 μg/mL), and incubated at 37°C in a humidified 5% CO_2_ incubator. At 24 h prior to the experiment, the culture medium was changed to DMEM supplemented with 10% charcoal-stripped FBS.

### 2.2 Reagents

Dulbecco’s Modified Eagle Medium (DMEM), FBS, penicillin, and streptomycin were purchased from GIBCO (Grand Island, NY). P4 was obtained from Sigma-Aldrich (St. Louis, MO); Anti-PR (sc-810) antibody was from Santa Cruz Biotechnology, Inc. (Santa Cruz, CA). Anti-cSrc (#2109), anti-p-cSrcY416 (#2101), anti-p-cSrcY527 (#2105), anti-Csk (#4980), anti-caveolin-1 (#3267), anti-p-caveolin-1 (#3251), anti-p140Cap (#3757) and anti-SHP2 (#9793) antibodies and SHP-2 siRNA (#7917) were purchased from Cell Signaling Technology Inc. (Beverly, MA). Anti-p-Csk antibody (#PA5-40214) was from Invitrogen (Carlsbad, CA). Antibody against G3PDH (GTX10011) was from GeneTex (Hsinchu, Taiwan).

### 2.3 Western blot analysis

Western blot analyses were performed as described previously (19). After incubation, the cells were washed with PBS (pH 7.4), incubated with extraction buffer (Tris 50 mM, pH 7.5, NaCl 150 mM, PMSF 1 mM, NP-40 1%, 0.1% SDS, 10 μg/mL Aprotinin and EDTA 10 mM) on ice, and then centrifuged at 12,000 ×g for 30 min. The cell extract was then boiled in a ratio of 3:1 with sample buffer (Tris–HCl 250 mM, pH 6.8, glycerol 40%, β-mercaptoethanol 20%, SDS 8% and bromophenol blue 0.04%). After electrophoresis in a 12% SDS–polyacrylamide gel (50 μg of protein for each lane), the separated proteins were then transferred onto PVDF membranes. The membrane was cut according to molecular weight with the help of protein marker prior to incubation with antibodies, pre-incubated with BSA (1%) and NaNO3 (0.02%) to block the non-specific IgGs, incubated with specific primary antibody (0.2 μg/mL) for 1 h at room temperature followed by horseradish peroxidase-conjugated second antibody (Jackson ImmunoResearch Laboratories, West Grove, PA) (1:10,000) for additional 1 h, and then developed using the enhanced chemiluminescence (ECL) system (GE, Healthcare, NJ). The intensity of each band was quantify using Image Pro-Plus 4.5 Software.

### 2.4 Co-immunoprecipitation

Co-immunoprecipitation was conducted as described previously (20). Briefly, protein (200 μg) was immunoprecipitated using primary antibody (2 μg) and protein A agarose beads (20 μL). The precipitates were then washed 5 times with lysis buffer and 1 time with PBS. The pellets were re-suspended in sample buffer (50 mM Tris, pH 6.8, 100 mM bromophenol blue, and 10% glycerol), incubated for 10 min at 90°C before electrophoresis to release the proteins from the beads, and vortexed in the centrifuge for 10 min at 12,500 rpm to discard the beads. The supernatant was fractionated by 8.5% SDS-PAGE, and the protein–protein interaction was examined using Western blot analyses.

### 2.5 Cell transfection

For transient transfection of SHP2 siRNA or/and ER siRNA into breast cancer cell lines, jetPEI transfection reagent (Polyplus Transfection, Bioparc, France) was used and the transfection was performed according to the manufacture’s protocol. Briefly, the cells were grown in the culture medium and then switched to transfection medium prior to siNRA transfection. The cell culture was treated with a mixture containing 6 μL of SHP2 siRNA or/and ER siRNA, 12 μL of jetPEI transfection reagent and 500 μL of transfection medium for 4-5 h. The transfection medium was removed, switched to DMEM containing 10% FBS for additional 24 h, and then rendered quiescent for 24 h using DMEM containing 0.04% FBS. The culture medium was switched to DMEM containing 10% FBS prior to P4 treatment. The SHP2 siRNA and ER siRNA were purchased from Cell Signal Technology (# 7917) and Genepharma (A10001-2099), respectively.

### 2.6 MTT assay

MTT was performed as previously described (18). Briefly, the cells were seeded onto 24-well dishes and grown in culture medium supplemented with 10% charcoal-stripped FBS. After the cells were treated with P4 (50 nM) for 96 h, the MTT reagent [3-(4,5-Dimethyl-2- thiazolyl)-2,5-diphenyl-tetrazolium bromide] (USB/Amersham Life Science, Cleveland, OH) at a concentration of 2.5 mg/mL was added to each well. The optical density (570 nM) was measured by ELISA reader. Four samples were analyzed in each experiment.

### 2.7 Wound healing assay

Wound healing assay was conducted as described previously (21). Briefly, when the cells grown in 12-well plates reached 100% confluence, 10 μL micropipette were used to scalp a “wound” in a monolayer of cells. The images captured at the beginning of wound healing assay were compared to the images after 12 h incubation of the cells in a humidified 5% CO_2_ incubator at 37°C. The cells migrating to close the wound were examined and counted under microscope to quantify the migration rate of the cells.

### 2.8 Statistical analysis

All data were expressed as the mean value ± s.e.mean. Comparisons were subjected to one way analysis of variance (ANOVA) followed by Fisher’s least significant difference test. Significance was accepted at *P* < 0.05.

## 3 Results

### 3.1 Involvement of SHP2 in the P4-induced cSrc activation in breast cancer cell lines

**Figure 1:**
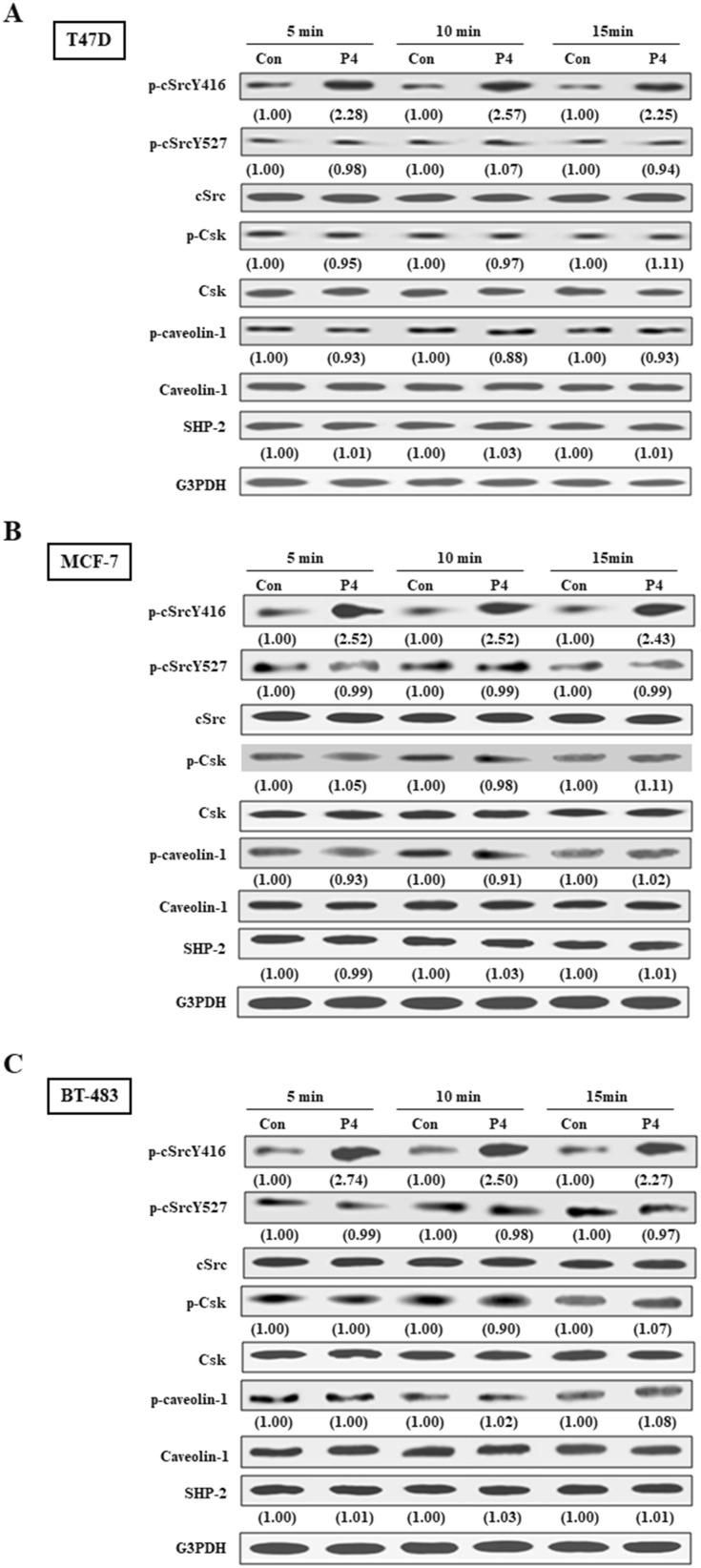
SHP2 contributes to P4-induced cSrc activation in breast cancer cell lines. Treatment with P4 (50 nM) for 5 min increased the level of p-cSrcY416, but did not significantly affect the levels of p-Csk and p-caveolin-1 protein in T47D **(A)**, MCF-7 **(B)**, and BT-483 **(C)** cell lines. Knockdown of SHP2 increased the levels of p-Csk and p-caveolin-1, and induced cSrc inactivation in the P4-treated T47D cells. The protein levels were examined by Western blot analyses and values shown in parentheses represent the quantified results adjusted with their own total protein or G3PDH and expressed as fold of control. Data are representative of 2 independent experiments with similar results. The gels have been run in the same experimental conditions and the cropped blots, which were cut prior to hybridization with antibodies, were shown. Con, control; NT siRNA, non-target small interfering RNA; siRNA, small interfering RNA.

We previously showed that P4 increased proliferation and migration of breast cancer cell lines through activating the cSrc-mediated signaling pathways (9, 10). In the present study, we further investigated the molecular mechanism underlying P4-regulated the cSrc-mediated signaling pathways. It has been suggested that high level of SHP2 in breast cancer cells might be involved in breast oncogenesis. Since we previously demonstrated that P4 (0-100 nM) concentration-dependently promoted the proliferation of breast cancer cell lines (T47D and MCF-7), and reached a plateau at 50 nM (9) that overlaps the physiologic range of plasma P4 concentrations in premenopausal women (22), we used P4 at 50 nM to investigate the role of SHP2 in the P4-induced cSrc activation. Using Western blot analysis and siRNA knockdown technique, we demonstrated that treatment with P4 (50 nM) for 5 min increased the levels of p-cSrcY416 (active form of cSrc) in T47D (**Figure 1A**), MCF-7 (**Figure 1B**) and BT-483 (**Figure 1C**) cell lines, and these effects were abolished by knockdown of SHP2. In contrast, knockdown of SHP2 increased the levels of p-cSrcY527 (inactive form of cSrc). Moreover, P4 increased the activities of cSrc negative regulatory proteins (p-Csk and p-caveolin-1) in these SHP2 knockdown breast cancer cell lines. These results suggest that SHP2 is required for the P4-induced cSrc activation in breast cancer cell lines.

### 3.2 Involvement of SHP2 in the P4-promoted proliferation and migration in breast cancer cell lines

**Figure 2:**
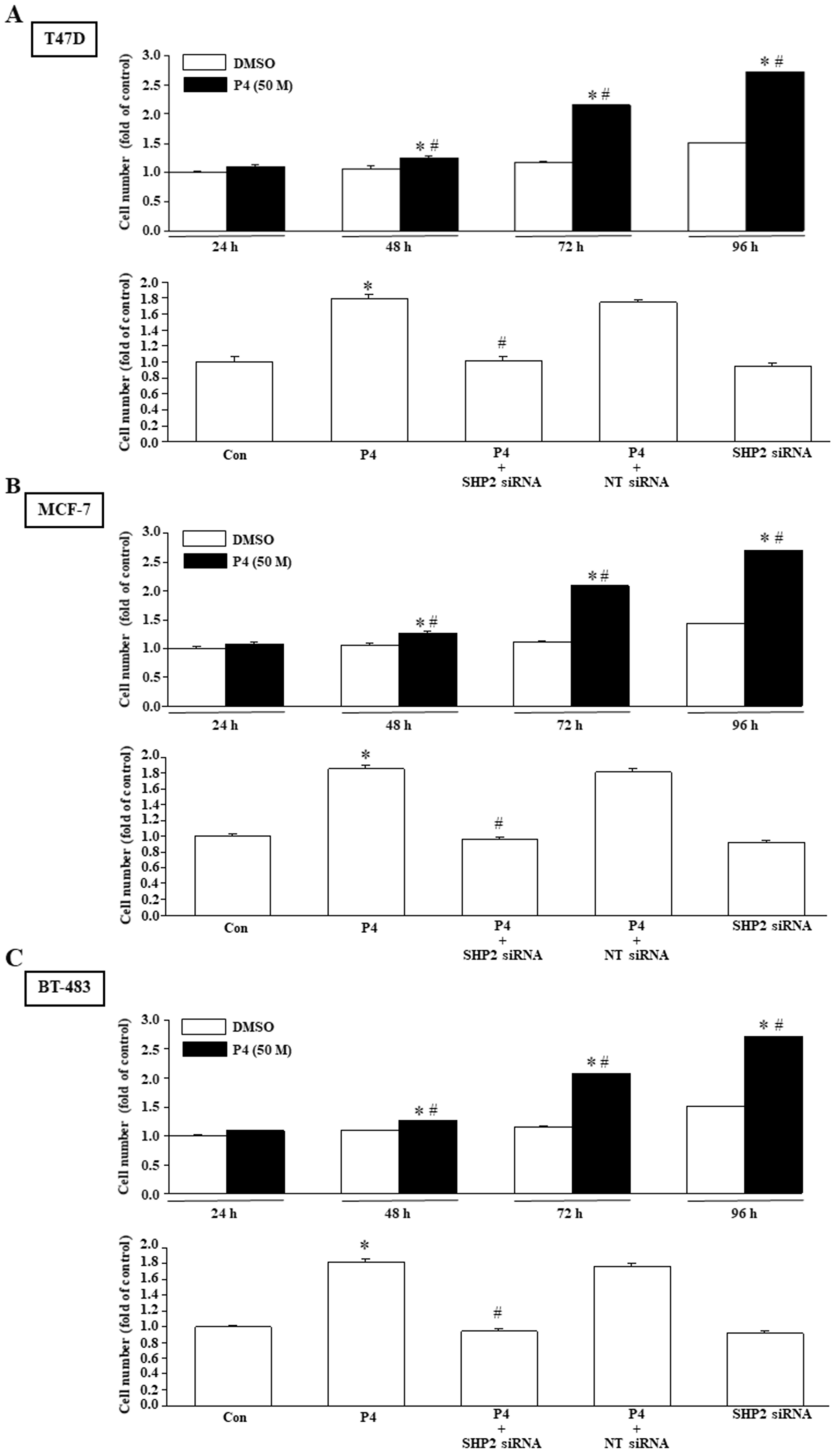
SHP2 is involved in the P4-promoted breast cancer cell proliferation. Treatment with P4 (50 nM) time-dependently promoted the cell proliferation in T47D (**A**, top panel), MCF-7 (**B**, top panel), and BT-483 (**C**, top panel). Knockdown of SHP2 using the siRNA technique prevented the P4-promoted proliferation in T47D (A, bottom panel), MCF-7 (**B**, bottom panel), and BT-483 (**C**, bottom panel). For proliferation assay, the cell number was measured using the MTT assay after daily treatment of the cell with P4 (50 nM) for three days. Values represent the means ± s.e.mean. (n = 4). ^*^P < 0.05 different from control. ^#^*P* < 0.05 different from the DMSO-treated group. NT siRNA, non-target small interfering RNA.

**Figure 3:**
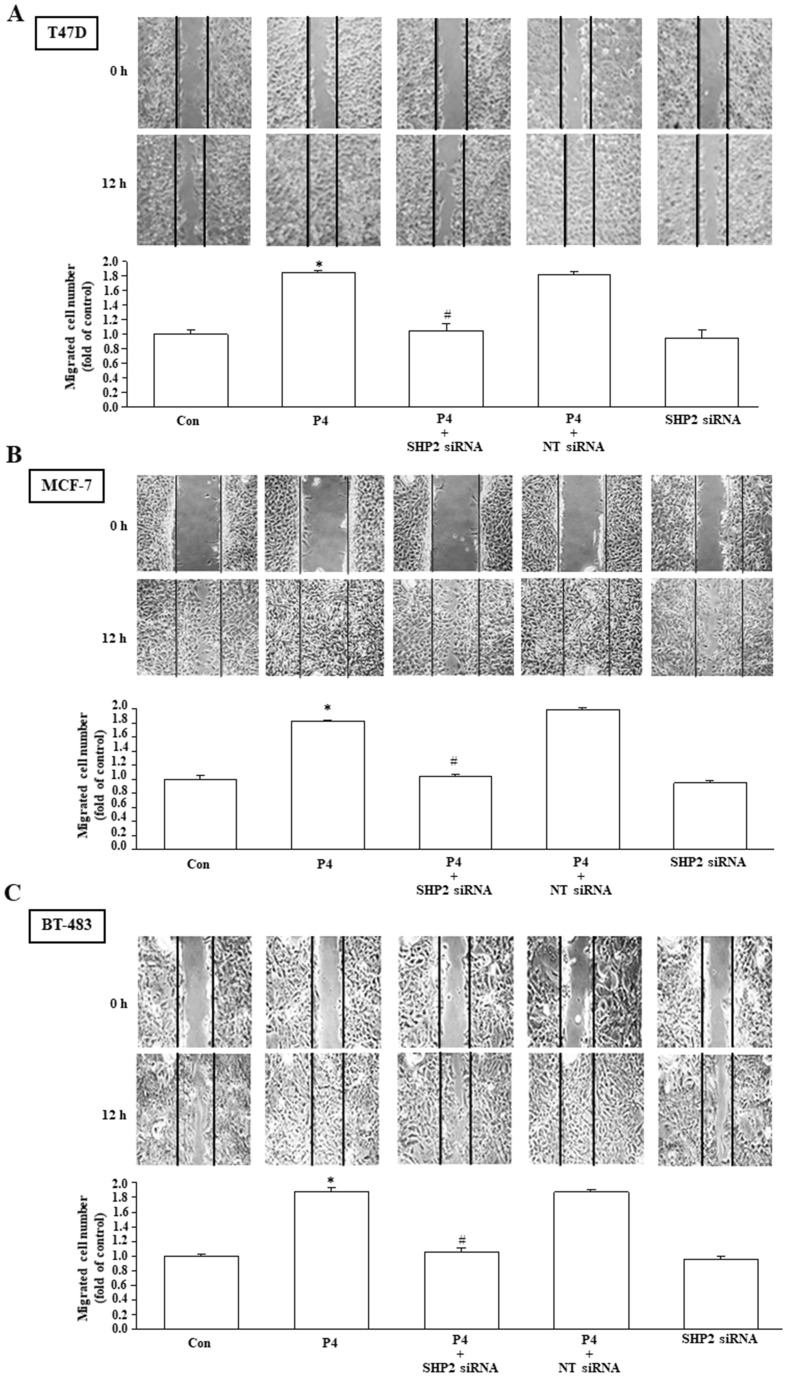
SHP2 is involved in the P4-promoted breast cancer cell migration. Knockdown of SHP2 using the siRNA technique prevented the P4-promoted migration in T47D **(A)**, MCF-7 **(B)**, and BT-483 **(C)**. Top pane: representative photographs of wound healing migration assay. Bottom panel: quantified results expressed by fold of control. For migration assay, the cells migrating to close the wound were counted to quantify the migration rate of the cells. Values represent the means ± s.e.mean. (n = 3). ^*^P < 0.05 different from control. ^#^*P* < 0.05 different from the P4-treated group. NT siRNA, non-target small interfering RNA.

Using MTT assay and wound healing migration assay, we investigated the effects of P4 treatment on breast cancer cell proliferation and migration. Our results show that P4 (50 nM) time-dependently increased the proliferation of T47D (**Figure 2A**, top panel), MCF-7 (**Figure 2B**, top panel) and BT-483 (**Figure 2C**, top panel). Knockdown of SHP2 expression abolished the P4-increased the proliferation of T47D (**Figure 2A**, bottom panel), MCF-7 (**Figure 2B**, bottom panel) and BT-483 (**Figure 2C**, bottom panel). P4 also increased the migration of T47D (**Figure 3A**), MCF-7 (**Figure 3B**) and BT-483 (**Figure 3C**) cell lines, and these effects were abolished by knockdown of SHP2 expression. These results suggest that SHP2 plays an essential role in the P4-increased proliferation and migration in breast cancer cell lines. Since three breast cancer cell lines (T47D, MCF-7 and BT-483) used in this study are classified as Luminal A estrogen receptor+ (ER+), PR+, HER2-, invasive ductal carcinoma, we tested whether these data are specific to this type of tumor by using ER knockdown T47D. Our data showed that double knocked-down of ER and SHP2 and single knocked-down of SHP2 did not show significantly different effects on the P4-increased T47D proliferation (**Supplementary Figure 1**) and migration (**Supplementary Figure 2**), suggesting that the requirement of SHP2 in the P4-promoted breast cancer cell proliferation and migration was not specific to ER+, PR+ breast cancer cells. Moreover, knocked-down of ER did not significantly affect the SHP2 siRNA-increased phosphorylation of Csk, caveolin and SrcY527 and the SHP2 siRNA-abolished P4-increased phosphorylation of Y416 (**Supplementary Figure 3**). These data suggest that the essential role of SHP2 on the P4-promoted breast cancer cell proliferation and migration is not specific to ER+, PR+, HER2- breast cancer cell lines.

### 3.3 Effects of SHP2 knockdown on the interaction among cSrc and cSrc regulatory proteins in T47D cell line

**Figure 4:**
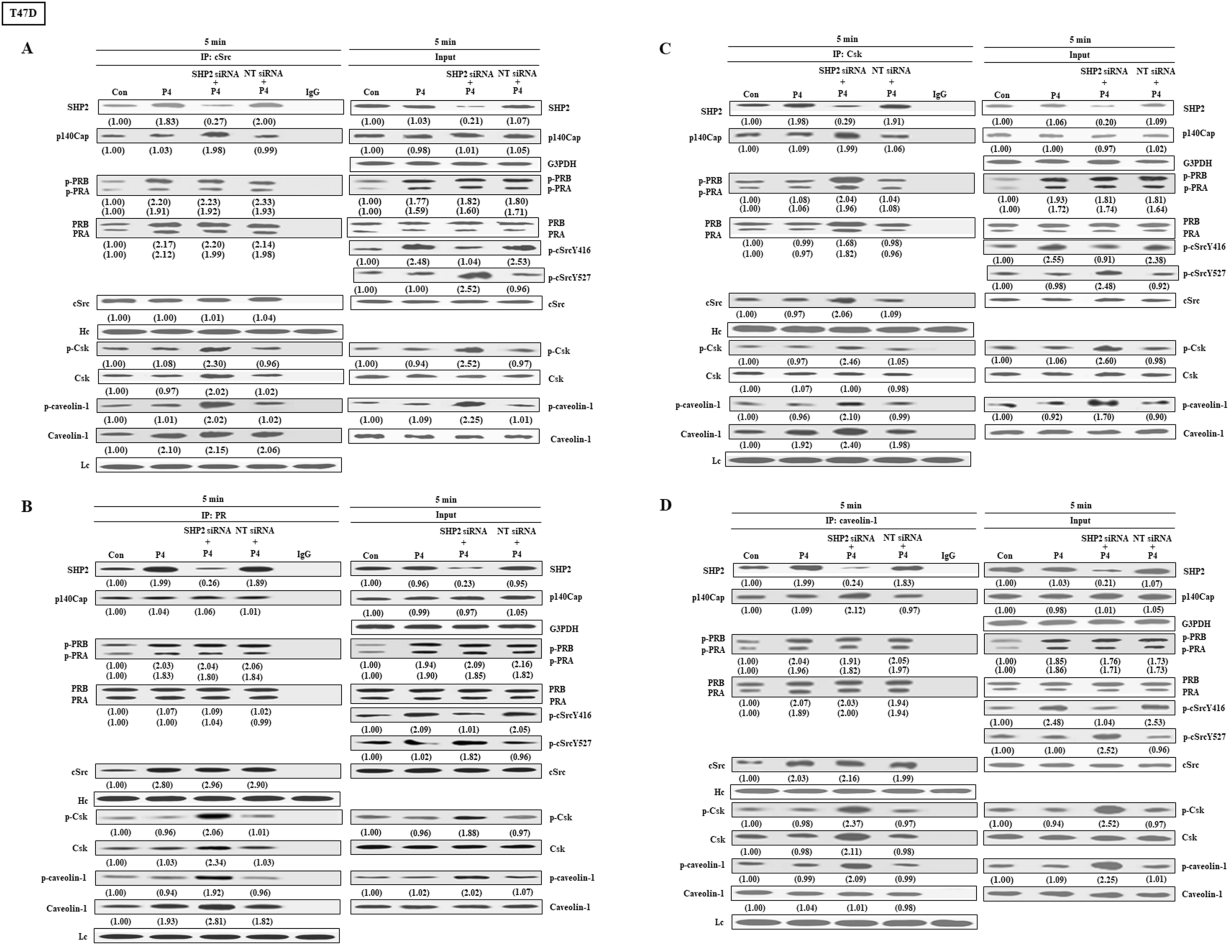
Knockdown of SHP2 increases the interaction between cSrc and cSrc-negative regulatory proteins in T47D cells. **(A)** Knockdown of SHP2 increased the levels of p-caveolin-1, p-Csk and p-cSrcY527, and the complex formations of cSrc-p-caveolin-1, cSrc-p140Cap, cSrc-Csk and cSrc-p-Csk in the P4-treated T47D cells, but did not affect the P4-increased complex formations of cSrc-PR and cSrc-p-PR. **(B)** Knockdown of SHP2 increased the interaction between caveolin-1 and cSrc-negative regulatory proteins (p140Cap and Csk), but did not affect the P4-increased complex formations of caveolin-1-PR and caveolin-1-p-PR. **(C)** Knockdown of SHP2 increased the complex formations of Csk-caveolin-1, Csk-p-caveolin-1, Csk-PR, Csk-p-PR, Csk-p140Cap and Csk-cSrc. The protein-protein interaction was detected by co-immunoprecipitation technique. The protein levels were examined by Western blot analyses and values shown in parentheses represent the quantified results adjusted with their own total protein or G3PDH and expressed as fold of control. Data are representative of 2 independent experiments with similar results. The gels have been run in the same experimental conditions and the cropped blots, which were cut prior to hybridization with antibodies, were shown. Con, control; NT siRNA, non-target small interfering RNA; siRNA, small interfering RNA.

**Figure 5:**
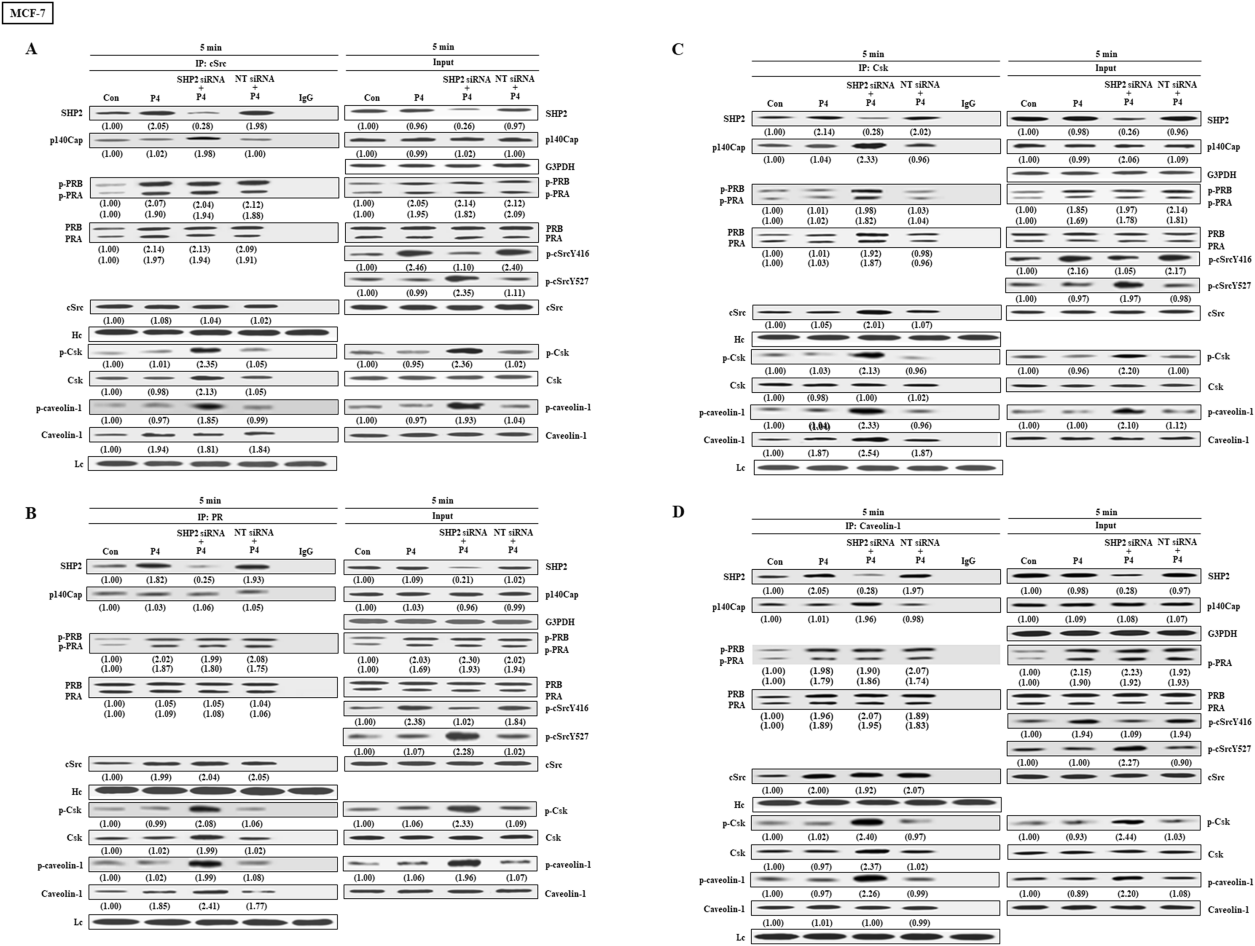
Knockdown of SHP2 increases the interaction between cSrc and cSrc-negative regulatory proteins in MCF-7 cells. **(A)** Knockdown of SHP2 increased the levels of p-caveolin-1, p-Csk and p-cSrcY527, and the complex formations of cSrc-p-caveolin-1, cSrc-p140Cap, cSrc-Csk and cSrc-p-Csk in the P4-treated MCF-7 cells, but did not affect the P4-increased complex formations of cSrc-PR and cSrc-p-PR. **(B)** Knockdown of SHP2 increased the interaction between caveolin-1 and cSrc-negative regulatory proteins (p140Cap and Csk), but did not affect the P4-increased complex formations of caveolin-1-PR and caveolin-1-p-PR. **(C)** Knockdown of SHP2 increased the complex formations of Csk-caveolin-1, Csk-p-caveolin-1, Csk-PR, Csk-p-PR, Csk-p140Cap and Csk-cSrc. The protein-protein interaction was detected by co-immunoprecipitation technique. The protein levels were examined by Western blot analyses and values shown in parentheses represent the quantified results adjusted with their own total protein or G3PDH and expressed as fold of control. Data are representative of 2 independent experiments with similar results. The gels have been run in the same experimental conditions and the cropped blots, which were cut prior to hybridization with antibodies, were shown. Con, control; NT siRNA, non-target small interfering RNA.

**Figure 6:**
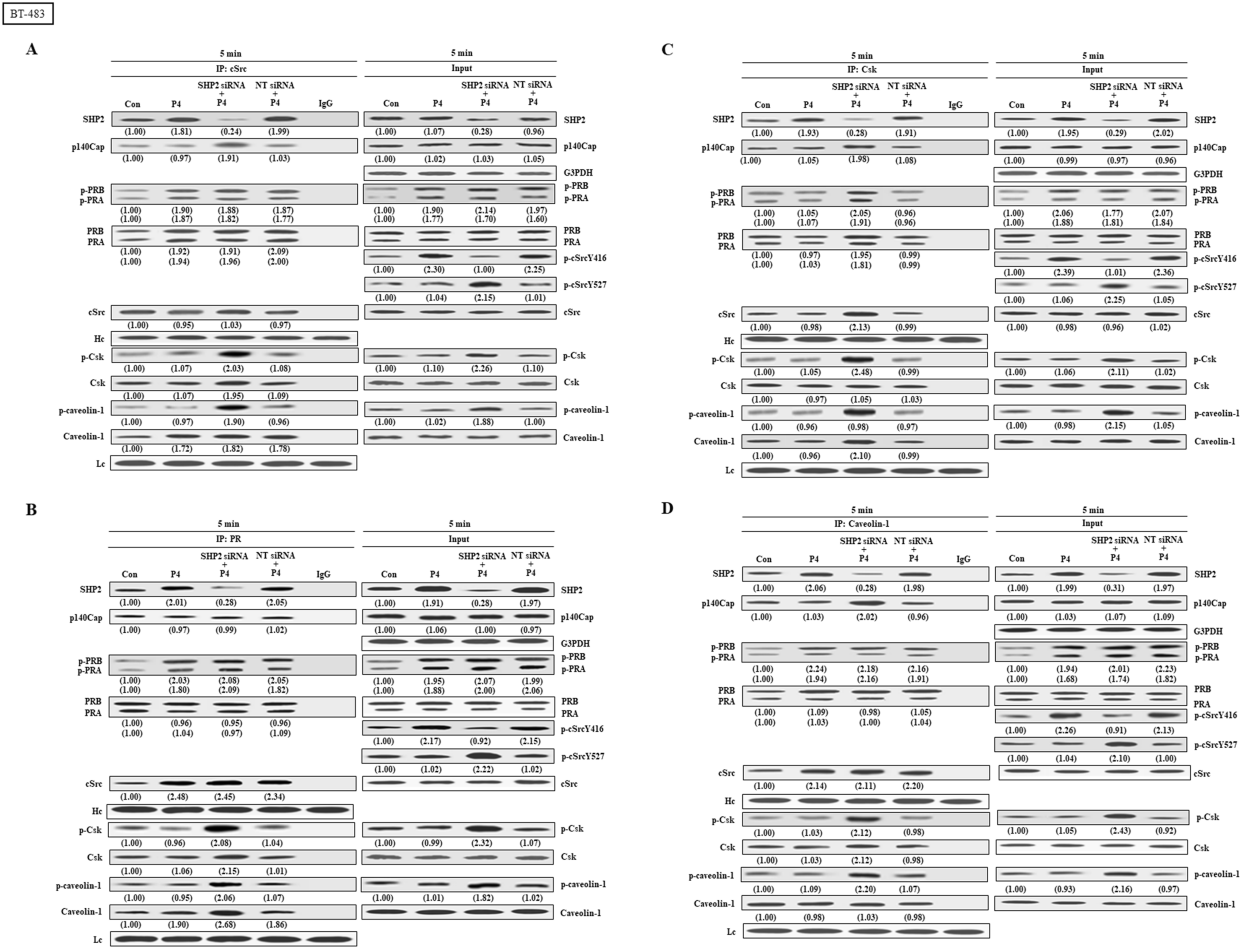
Knockdown of SHP2 increases the interaction between cSrc and cSrc-negative regulatory proteins in BT-483 cells. **(A)** Knockdown of SHP2 increased the levels of p-caveolin-1, p-Csk and p-cSrcY527, and the complex formations of cSrc-p-caveolin-1, cSrc-p140Cap, cSrc-Csk and cSrc-p-Csk in the P4-treated BT-483 cells, but did not affect the P4-increased complex formations of cSrc-PR and cSrc-p-PR. **(B)** Knockdown of SHP2 increased the interaction between caveolin-1 and cSrc-negative regulatory proteins (p140Cap and Csk), but did not affect the P4-increased complex formations of caveolin-1-PR and caveolin-1-p-PR. **(C)** Knockdown of SHP2 increased the complex formations of Csk-caveolin-1, Csk-p-caveolin-1, Csk-PR, Csk-p-PR, Csk-p140Cap and Csk-cSrc. The protein-protein interaction was detected by co-immunoprecipitation technique. Western blot analyses were conducted to examine the protein levels. Values shown in parentheses represent the quantified results adjusted with their own total protein or G3PDH and expressed as fold of control. Data are representative of 2 independent experiments with similar results. The gels were run in the same experimental conditions and the cropped blots, which were cut prior to incubation with antibodies, were shown. Con, control; NT siRNA, non-target small interfering RNA.

We further examined the effects of SHP2 knockdown on the interaction among cSrc and its regulatory proteins in breast cancer cell lines (T47D, MCF-7 and BT-483). Co-immunoprecipitation assay demonstrated that treatment with P4 increased the complex formations of cSrc-SHP2, cSrc-PRA, cSrc-PRB, and cSrc-cavolin-1, PR-SHP2, and PR-caveolin-1, and the levels of p-PRA, p-PRB, and p-cSrcY416, but did not significantly affect the complex formations of cSrc and cSrc-negative proteins (Csk and p140Cap) in breast cancer cell lines, and the levels of p-Csk, p-caveolin-1, and p-cSrcY547 (inactive form of cSrc). In the SHP2 knockdown breast cancer cell lines, however, the P4-increased p-cSrcY416 level was abolished, and P4 increased the levels of p-caveolin-1, p-Csk and p-cSrcY527 (**Figures 4A–D**, **5A–D**, **6A–D**), PR-cSrc negative regulatory proteins (**Figures 4B**, **5B**, **6B**), Csk-PRA, Csk-PRB, Csk-p-PRA, Csk-p-PRB, and Csk-p140Cap (**Figures 4C**, **5C**, **6C**), caveolin-1-Csk, and caveolin-1-p140Cap (**Figures 4D**, **5D**, **6D**). The P4-increased complex formations of cSrc-caveolin-1, cSrc-PRA and cSrc-PRB were not affected by knockdown of SHP2. Although P4 increased the complex formation of cSrc-cavolin-1, the level of p-caveolin-1 was not affected. However, P4 increased the level of p-cavolin-1 in the SHP2 knockdown breast cancer cell lines.

### 3.4 Role of PR in the P4-increased formation of cSrc-SHP2 complex

We also examined the effect of PR knockdown on the P4-induced formation of c-Src-SHP2 complex. Co-immunoprecipitation assay demonstrated that in the absence of P4, knockdown of PR abolished the formation of cSrc-SHP2 complex in T47D cell line. Moreover, the P4-increased complex formations of cSrc-SHP2 in T47D cell line was also abolished by knockdown of PR (**Supplementary Figures 4A, B**). These findings suggest that PR is essential for the formation of cSrc-SHP2 complex.

## 4 Discussion

We previously demonstrated that P4 increased the proliferation (9) and migration (10) in breast cancer cell lines through activating the cSrc-mediated signaling pathways. However, the molecular mechanism underlying P4-induced cSrc activation is still unclear. Since SHP2 is abundant in breast cancer cells and has been suggested to be involved in breast oncogenesis (23), we investigated the possible contribution of SHP2 in the P4-promoted breast cancer cell proliferation and migration. Our data showed that knockdown of SHP2 abolished the P4-induced cSrc activation and enhancement of proliferation and migration in breast cancer cell lines. To our knowledge, this was the first demonstration that the presence of SHP2 is essential for the P4-promoted breast cancer cell proliferation and migration.

Progestins have also been shown to induce a direct interaction between the polyproline motif of PR-B and the SH3 domain of cSrc, which results in cSrc activation in T47D cells (24). We previously demonstrated that P4 increased the complex formation of PR-cSrc and phosphorylation of cSrc at tyrosine 416 in breast cancer cell lines (14, 18). In the present study, we showed that treatment of T47D cells with P4 increased the levels of p-cSrcY416, but did not significantly affect the levels and activities of cSrc-negative regulatory proteins in T47D, MCF-7 and BT-483. However, the interaction between SHP2 and cSrc as well as cSrc-negative regulatory proteins were increased in the P4-treated T47D, MCF-7, and BT-483 breast cancer cell lines.

SHP2 contains two SH2 domains (N-SH2 and C-SH2), a PTP catalytic domain and a C-terminal tail (25, 26). In the basal condition, SHP2 is kept in an auto-inhibited conformation by intramolecular interactions between the N-SH2 domain and the catalytic cleft of the PTP domain (27). Upon stimulation, binding of growth factor receptor or adaptor proteins to the N-SH2 domain releases auto-inhibition, hence activating SHP2. SHP2 also contains proline-rich domain, which can bind to the SH3 domain of cSrc (28). It has been indicated that SHP2 can function as a positive regulator in cSrc signaling by interfering with the Csk-caveolin-1 complex formation in the H_2_O_2_-treated astrocytes (14). SHP2 overexpression is commonly observed in HER2 (+) ERα⁄PR (+) infiltrating ductal carcinoma, and the elevated level of SHP2 protein in breast cancer is positively correlated with lymph node metastasis and higher tumor grade (23). Knockdown of SHP2 in established breast cancers reduced their growth and metastasis (15). In the present study, we showed that treatment of T47D, MCF-7 and BT-483 cells with P4 increased the complex formations of cSrc-PR, cSrc-SHP2, and cSrc-caveolin-1. The interaction between SHP2 and cSrc-negative regulatory proteins (p140Cap, caveolin-1 and Csk) was also increased by P4 treatment. However, the levels of p-caveolin-1 and p-cSrc were not significantly increased by P4 treatment. These data suggest that SHP2 could prevent the interaction between cSrc and cSrc-negative regulatory proteins, hence causing the cSrc activation.

PR has been shown to directly bind to SH3 domain of c-Src through PR polyproline domain at the N-terminus and directly activate c-Src without dephosphorylation at tyrosine 527 (26). Treatment with P4 increased the formation of cSrc-PR complex and activated PR. These effects were not affected by knockdown of SHP2. However, P4 increased the formation of PR-Csk and PR-caveolin-1 in the SHP2 knockdown breast cancer cell lines. Our data suggest that the presence of SHP2 prevents the phosphorylation of cSrc at tyrosine 527, and P4 can directly activate cSrc without dephsophorylation at tyrosine 527. Knockdown of SHP2 increased the levels of p-caveolin-1 and p-Csk protein in T47D, MCF-7 and BT-483, and the complex formations of cSrc-cSrc negative regulatory proteins (p-caveolin-1 and p-Csk) in the P4-treated T47D cells. These data suggest that knockdown of SHP2 activated caveolin-1, subsequently enhancing the complex formation of cSrc-cSrc negative regulatory proteins, thereby increasing the phosphorylation of cSrcY527 and causing cSrc inactivation.

Caveolins, a family of integral membrane proteins, are the principal components of caveolae membrane and involved in regulation of a number of signaling pathways including those involved in cell proliferation, migration and transformation, and vesicular transport (29, 30). Caveolin-1 can act as a docking protein for both Csk and SHP2. It has been demonstrated that SHP2 can bind to caveolin-1 and contribute to the activation of cSrc in the H_2_O_2_-treated astrocytes through the competitive binding of Csk to caveolin-1 (14). In breast cancer cell lines, P4 can increase the complex formation of SHP2-caveolin-1, which prevented the P4-increased formations of Csk-p-caveolin-1 and Csk-cSrc) complex, hence increasing the phosphorylation of cSrcY416 and causing cSrc activation. Knockdown of SHP2 increased the phosphorylation of cSrc-negative regulatory proteins (caveolin-1 and Csk) and the complex formations of cSrc-p140Cap, cSrc-Csk, caveolin-1-p140Cap, and Csk-p140Cap, thereby increasing the phosphorylation of cSrcY527 and causing cSrc inactivation. We previously demonstrated that P4 promoted breast cancer cell proliferation (9) and migration (10) through activating the P4 receptor (PR)/cSrc-mediated signaling pathways. Taken together, knockdown of SHP2 prevented the P4-increased cSrcY416 and increased the level of cSrcY527 in the P4-treated breast cancer cell lines, suggesting that the presence of SHP2 played an important role in the P4-promoted breast cancer cell proliferation and migration.

p140Cap is an adaptor protein, and its carboxy-terminal domain contains a proline-rich sequence, which can bind to the cSrc SH3 domain, hence reducing the activity of cSrc (31, 32). Deletion of this proline-rich domain carboxy-terminal, the inhibition of cSrc activity induced by p140Cap mutants disappears (31). These results suggest that p140Cap can directly bind to cSrc, hence reducing the cSrc kinase activity. The p140Cap-caused inhibition of cSrc kinase activity is mediated by elevating the Csk phosphorylation. Csk, an endogenous regulator of the cSrc activity, can increase phosphorylation of the inhibitory Tyr527 in the cSrc carboxy-terminal domain, thereby allowing the binding of the cSrc SH2 domain and the acquirement of an inactive, closed conformation (33, 34). p140Cap was demonstrated to be able directly bind to Csk (33) and p140Cap overexpression can inhibit cell proliferation via reducing the cSrc kinase activity caused by Csk activation. In contrast, knockdown expression of p140Cap can enhance the activity of cSrc kinase (35). The complex formation of p140Cap-Csk contributes to the p140Cap-regulated cSrc inactivation. In the present study, we showed that knockdown of SHP2 increased the complex formations of cSrc-cSrc negative regulatory proteins (Csk and caveolin-1) and Csk-p140Cap, thereby increasing phosphorylation of cSrc at tyrosine 527 and cSrc inactivation.

In conclusion, the results from this study suggest that P4 increased the complex formation of PR-cSrc-SHP2, which increased the binding between SHP2 and caveolin-1, subsequently reducing complex formation of cSrc-Csk and cSrc-p140Cap, leading to decreased phosphorylation of cSrc527 and prolonged cSrc activation, and eventually promoted breast cancer cell proliferation and migration. Knockdown of SHP2 increased the complex formations of cSrc-Csk, cSrc-p140Cap and cSrc-p-caveolin-1, subsequently inducing phosphorylation of cSrc at tyrosine 527 and inactivation of cSrc, and eventually abolished the P4-promoted breast cancer proliferation and migration.

## Data Availability

The datasets presented in this study can be found in online repositories. The names of the repository/repositories and accession number(s) can be found in the article/**Supplementary Material**.
