# Supplementary material for: SHP2 is essential for the progesterone-promoted proliferation and migration in breast cancer cell lines
Source: Front Endocrinol (Lausanne). 2025 Feb 10;16:1523589. doi: 10.3389/fendo.2025.1523589 (PMC11847685; doi:10.3389/fendo.2025.1523589)
Supplement: Supplementary file 1 [file DataSheet1.pdf]

SHP2 is essential for the progesterone-promoted proliferation and migration in breast cancer cell lines

Hui-Chen Wang[1,2] and Wen-Sen Lee[1,3,4,5*]
[1]Graduate Institute of Medical Sciences, College of Medicine, Taipei Medical University, Taipei 110, Taiwan
[2]Second Degree Bachelor of Science in Nursing, College of Medicine, National Taiwan University, Taipei, Taiwan
[3] Taipei Cancer Center, Taipei Medical University Hospital, Taipei 110, Taiwan
[4]Cell Physiology and Molecular Image Research Center, Wan Fang Hospital, Taipei Medical University
[5]Department of Physiology, School of Medicine, Tzu Chi University, Hualien 97004, Taiwan

Supplemental Figure Legends

Suppl. Figure 1. Knockdown of ER does not significantly affect the P4-increased the proliferation of T47D cell line. Top panel shows representative results of ER, SHP2, and G3PDH protein levels detected by Western blot analysis. Bottom panel shows the quantitative results of cell number measured by MTT assay after treatment with P4 or vehicle for 96 h and expressed by fold of control. Values represent the means ± s.e.mean. (n = 3). * $P < 0.05$ different from the ER siRNA+vehicle group. ER, estrogen receptor; P4, progesterone; NT, non-target siRNA.

Suppl. Figures 2. Knockdown of ER does not significantly affect the P4-increased the migration of T47D cell line. Top panel shows representative results of ER, SHP2, and G3PDH protein levels detected by Western blot analysis. Middle panel shows representative photographs of wound healing migration assay. Bottom panel shows the quantitative results of migrated cell number measured by cell counting under microscope after treatment with P4 or vehicle for 12 h and expressed by fold of control. Values represent the means ± s.e.mean. (n = 3). * $P < 0.05$ different from the ER siRNA+vehicle group. ER, estrogen receptor; P4, progesterone; NT, non-target siRNA.

Suppl. Figures 3. ER is not involved in the P4-activated cSrc. Top panel shows representative results of ER, and G3PDH protein levels detected by Western blot analysis. Bottom panel shows that knocked-down of ER did not significantly affect the SHP-2 siRNA-increased phosphorylation of Csk, caveolin and SrcY527 and the SHP-2 siRNA-abolished P4-increased phosphorylation of Y416. The protein levels were examined by Western blot analyses and values shown in parentheses represent the quantified results adjusted with their own total protein or G3PDH and expressed as fold of control. ER, estrogen receptor; P4, progesterone; NT, non-target siRNA.

Suppl. Figure 4. PR is essential for the formation of cSrc-SHP2 complex. Knockdown of PR using the PR siRNA technique abolished the formation of cSrc-SHP2 complex detected by cSrc IP (A) and SHP2 IP (B). IP, immunoprecipitation; P4, progesterone; NT, non-target siRNA.

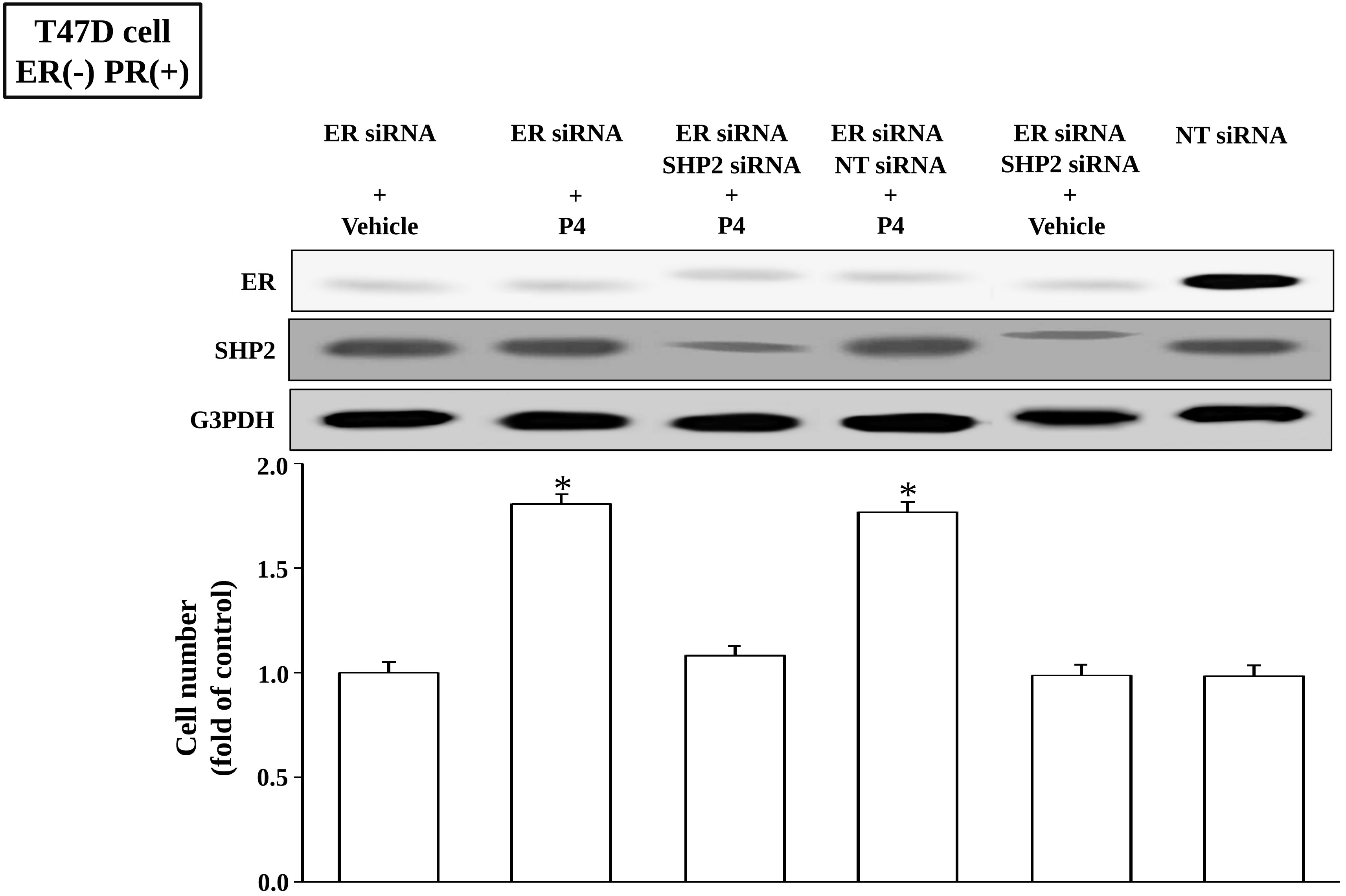

Suppl. Figure 1

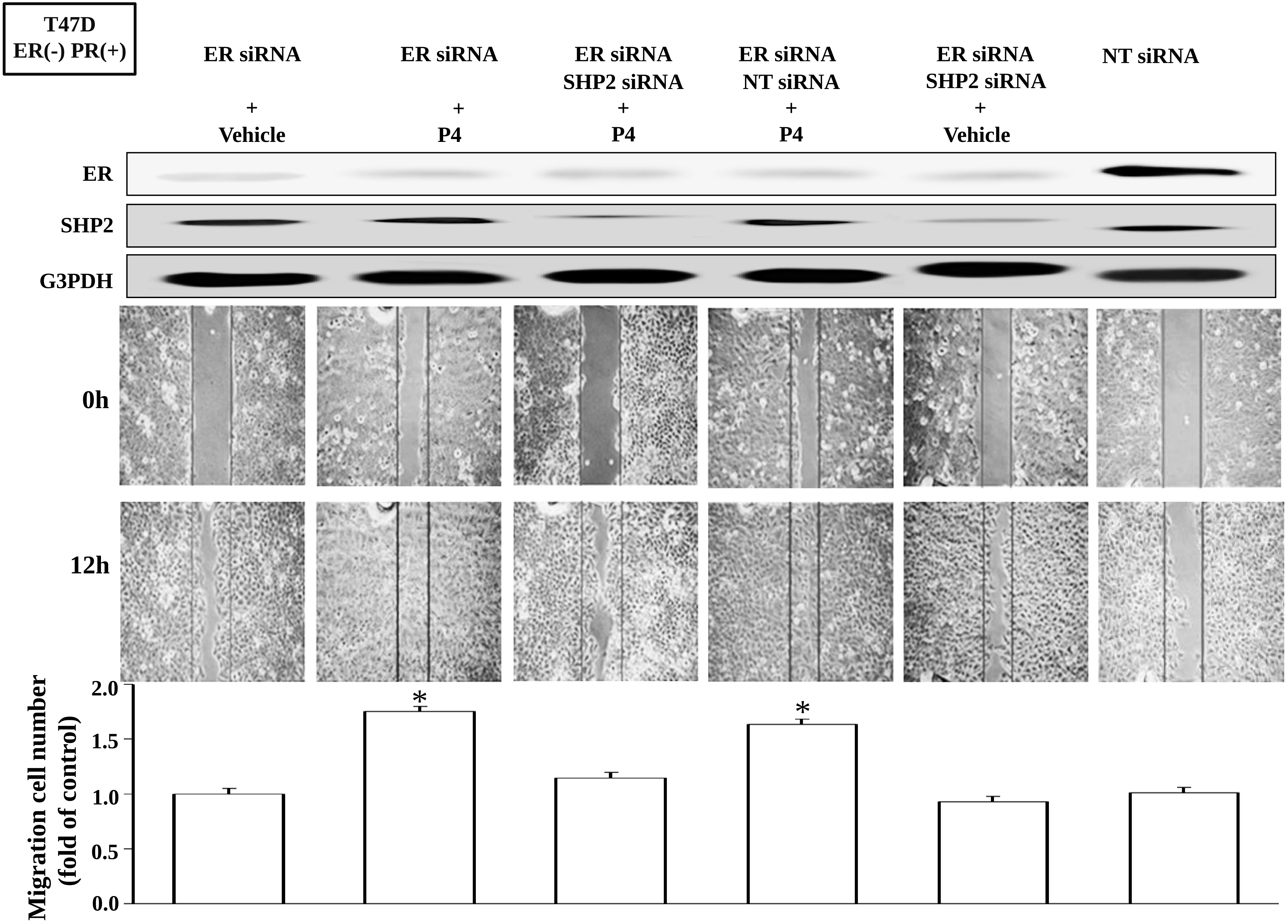

Suppl. Figure 2

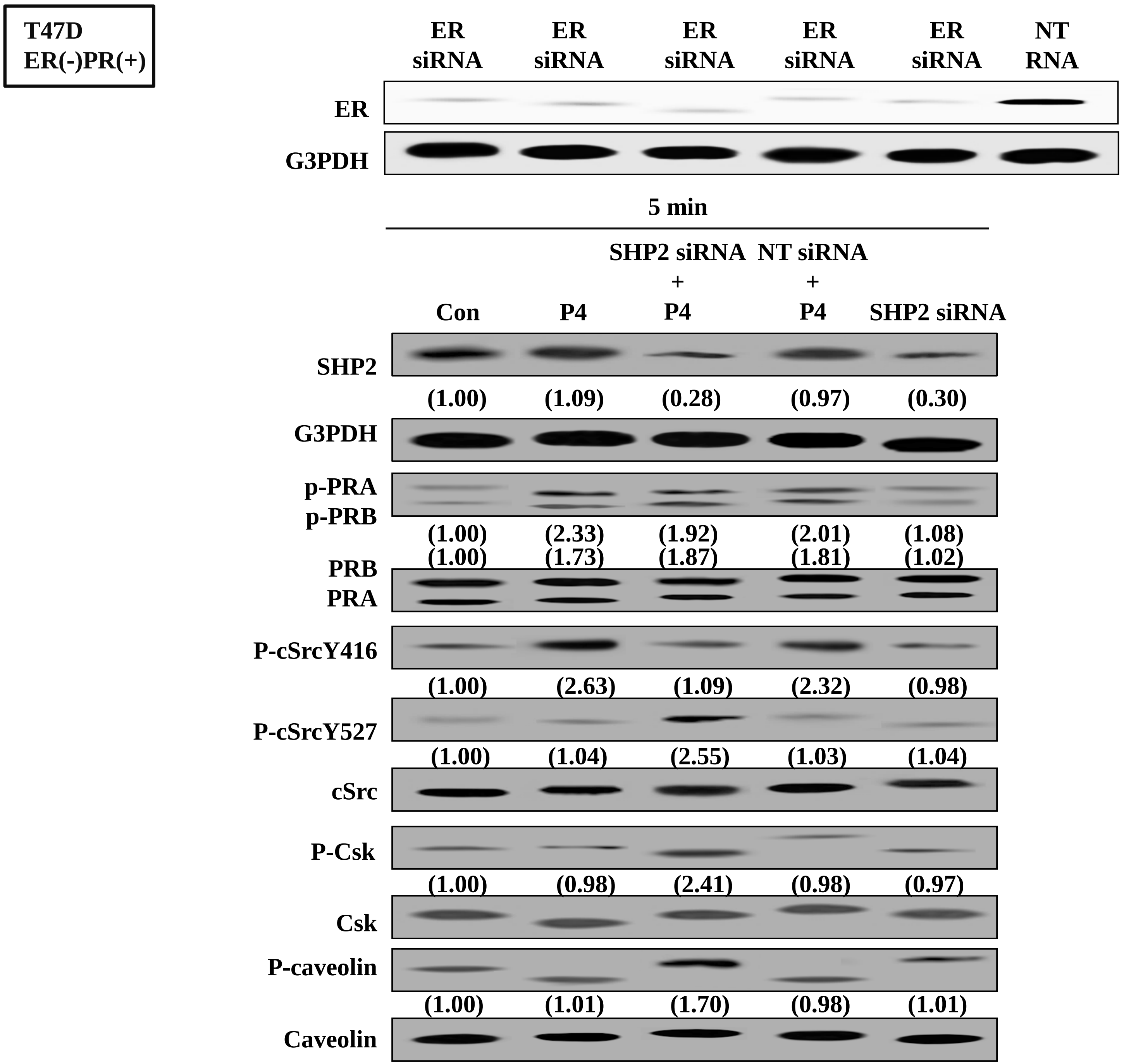

Suppl. Figure 3

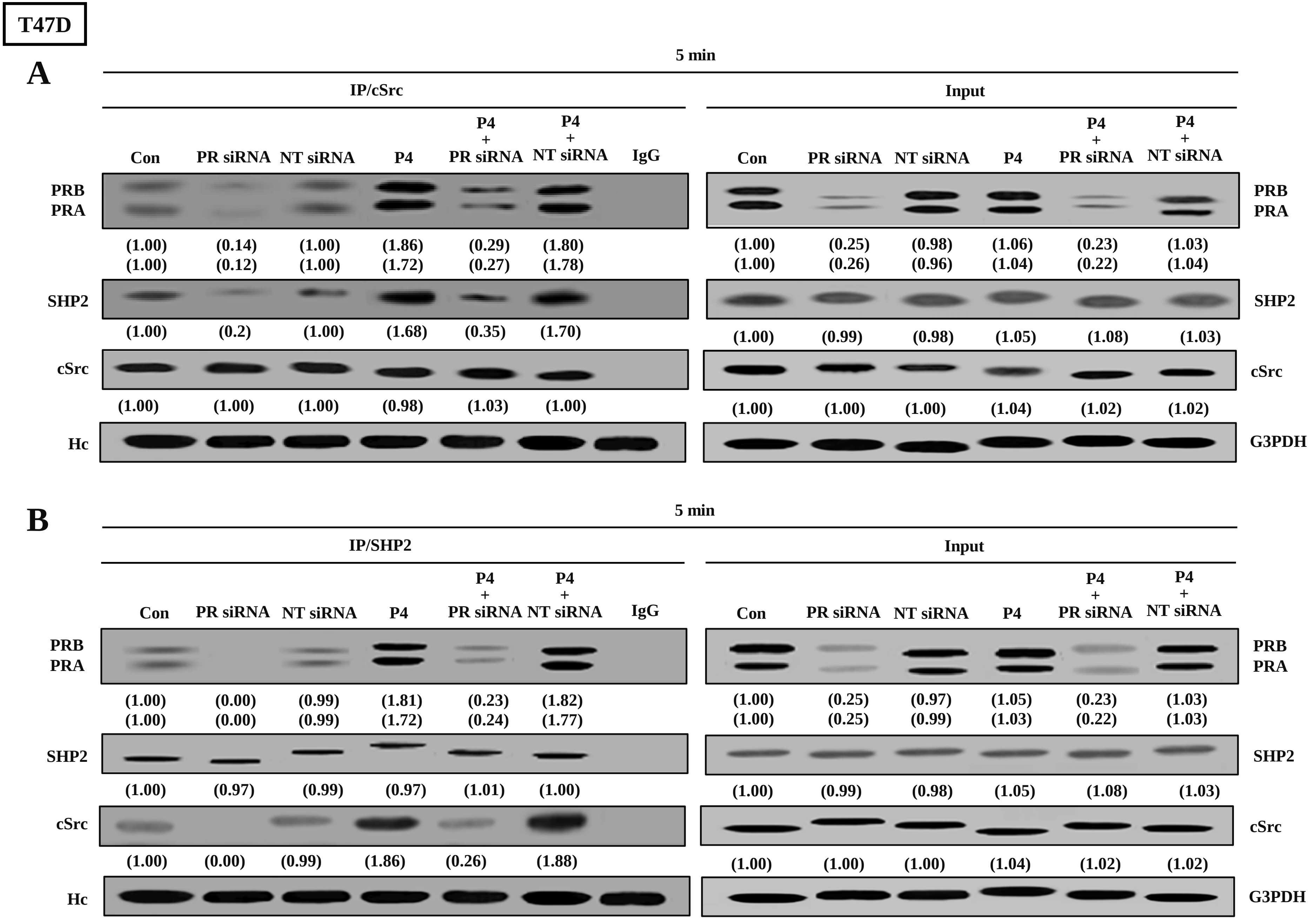

Suppl. Figure 4
